# Neonatal and pediatric nutritional care: a modified Delphi consensus on dietary management for infants with congenital heart disease in four Middle Eastern countries

**DOI:** 10.3389/fped.2026.1881464

**Published:** 2026-09-30

**Authors:** Alyaa Kotby, Ahmed Adel, Banseh Ahmed Hussein, Ensaf Boraey Othman Moustafa, Fahd Al-Harby, Hesham Samir Hasan Moustafa, Mahmoud Al-Husseiny, Mahmoud Al-Soufi, Manal Assiri, Masouma Al-Gasmi, Mohamed Hussein Metwally, Mohamed Matter, Mohamed Samy Al Farsy, Rabih Khalil Abdul Rahim, Rania Abdelkarim

**Affiliations:** 1 Pediatric Department, Cardiology Division, Ain Shams University, Cairo, Egypt; 2 Al Jalila Children’s Specialty Hospital, Dubai Health, Dubai, United Arab Emirates; 3 Dar Al Shifa Hospital, Hawally, Kuwait; 4 Dr Sulaiman Alhabib Medical Group, Women’s Health Hospital, Riyadh, Saudi Arabia; 5 Neonatal Intensive Care Unit, New Jahra Hospital, Al Jahra, Kuwait; 6 Neonatal Intensive Care Unit, King Abdulaziz Hospital, Jeddah, Saudi Arabia; 7 Pediatrics and Neonatology Department, Ain Shams University, Cairo, Egypt; 8 Pediatric Department, Mansoura University, Mansoura, Egypt; 9 Pediatrics Department, Faculty of Medicine, Ain Shams University, Cairo, Egypt; 10 Alorf Hospital, Al Jahra, Kuwait

**Keywords:** congenital heart disease, Delphi consensus, infants, malnutrition, nutrition therapy, The Middle East

## Abstract

**Background:**

In the absence of localized guidelines for the Middle East region, management of infant malnutrition secondary to congenital heart disease (CHD) can lead to suboptimal outcomes and may aggravate patient conditions. In this consensus document, we aimed to guide health care professionals on care pathways and establish regionally relevant recommendations for the nutritional assessment and management of infants with CHD.

**Methods:**

Fifteen experts from four Middle Eastern countries participated in a two-round modified Delphi process. The included statements were initially developed following a literature review and subsequently refined based on feedback from a steering committee of five experts preselected from the panelists’ group for the first voting round. The second voting round, involving the other ten panelists, determined the final voting results. Follow-up discussions were utilized when needed. Consensus was predefined as ≥80% agreement, and statements meeting this threshold were included in the final document.

**Results:**

Consensus was reached with a total of 43 statements, representing 87.8% of the suggested statements. The consensus statements established essential components and cut-off points for diagnosing malnutrition, specified values for enteral and parenteral feeding, caloric and fluid requirements and management, feeding practices and use of formula, and care in acute and critical settings. The highest agreement level was associated with the perioperative nutrition recommendations, while nutritional support recommendations faced the largest variability in response. In the latter, areas where a heterogeneity in clinical practice was observed, reflected by the lack of consensus, were identified.

**Conclusion:**

This consensus document provides a general framework for the assessment, management, and follow-up of infants with CHD with compromised nutritional status in four Middle Eastern countries, bridging the gap between existing international guidelines and real-world clinical practice.

## 1 Introduction

Congenital heart disease (CHD) has been associated with an increased risk of malnutrition in neonates and infants; the magnitude of which varies by the type of CHD, associated conditions, cardiac complications, and genetics (1). The overlap between malnutrition and CHD is particularly important in the case of newborns and infants, where the vicious cycle of increased energy expenditure, inadequate absorption, and decreased feeding results in cognitive and physical growth impairments during this period of critical growth and development (1–4).

Estimates for the prevalence of malnutrition in infants with CHD vary in the literature. However, prevalence rates as high as 84% are reported, with severe malnutrition being present in up to 71% of those cases (5). On the other hand, a systematic review reported a range between 15% and 64% for malnutrition in CHD (6). Malnutrition and failure of growth are also risk factors for infant mortality in CHD (7). A decrease in weight for age and height for age Z scores was associated with 40% and 33% higher mortality, respectively (8).

The Middle East region has been reported to have the highest rates of birth defects (9); this is particularly evident in the high reported CHD incidence and prevalence in Middle Eastern high-income countries (10, 11), while such high rates are globally limited to low- and middle-income countries (12). There are no epidemiological data on CHD in the Middle East as a whole, but data from scattered studies estimate an incidence between 2.1 and 14.8 per 1,000 live births (13–17).

Despite the sustained trend of the exceptionally high prevalence of CHD in this region, and the abundance of research regarding CHD management in the Arab world (18), there is still a lack of a defined framework for malnutrition detection, management, and prevention that is tailored to this population. This is further complicated by the multifactorial nature of malnutrition in patients with CHD (1). Hence, enhancing the knowledge of the involved health care practitioners (HCPs) on CHD pathological features and hemodynamics is essential, where successful catch-up growth can only be achieved when nutritional rehabilitation is combined with optimal cardiac treatment. Additionally, the lack of standardization in practices regarding optimal nutritional support for infants with CHD would likely aggravate the poor growth of those infants, fueled by inadequate or delayed interventions, suboptimal levels of care, and poor allocation of resources.

Accordingly, this consensus document aims to guide pediatricians and general practitioners in this region on how to assess, manage, and follow up infants with CHD and compromised nutritional status. Moreover, streamlining nutritional care will also help doctors support family caregivers and ease their role in their infant’s disease journey, where feeding remains the greatest challenge for these families in the early years of the infant’s life (19, 20). Similar efforts were made in different regions using Delphi consensus methodology to standardize nutritional care in infants with CHD, where the heterogeneity and variability in nutritional practices have been widely recognized in the past years, as well as their clinical consequences (21, 22). However, these frameworks may not have fully reflected the clinical characteristics, availability of resources, and other variations in care across the Middle East. Consequently, there remains a need for region-specific consensus recommendations that account for local practice patterns.

## 2 Materials and methods

### 2.1 Panel composition

This consensus, expert opinion-based document was developed via a modified Delphi approach in which a total of 15 panelists participated. The panelists were classified into two groups: (1) the steering committee, which is a group of five specialists (a pediatrician, a neonatologist, two pediatric cardiologists, a nutritionist) from four Middle Eastern countries [Egypt, Kuwait, Saudi Arabia [KSA], and United Arab Emirates [UAE]] representing the main advisors in this consensus process, (2) a second group of ten practitioners from the same specialties as Group 1.

### 2.2 Questionnaire development

The survey questions were developed with the support of an external research consultancy from a contract research organization (CRO); more details on the involvement of external support are described in the acknowledgements section. The first group of experts engaged in a brainstorming exercise, and a non-systematic literature review was performed by the scientific team appointed by the CRO to identify the main topics to be covered. PubMed, EMBASE, and The Cochrane Library databases were searched for articles published between 2010 and 2024. The literature search took place in April 2024 and continued till July 2024. We used the keywords: Congenital Heart Disease, Congenital Heart Defects, CHD, Congenital Cardiac Anomalies, Infant, Newborn, Neonates, Preterm Infants, Full-Term Infants, Nutrition Assessment, Nutritional Status, Dietary Assessment, Growth Monitoring, Anthropometry, Nutrition Therapy, Dietary Management, Nutritional Support, Enteral Nutrition, Parenteral Nutrition, Malnutrition, Undernutrition, Growth Failure, Failure to Thrive, Caloric Needs, Breastfeeding, and Formula Feeding, to identify the relevant articles. We included original articles, systematic and narrative review articles, and guidelines. We prioritized research originating from the Middle East region without restricting our literature review to these articles only. Relevant information was then extracted from the retrieved articles and was used to draft the initial statements. The initial form of the survey was then developed based on the results of the above-mentioned data mining process.

The included core topics (CHD nutritional risk, nutritional assessment, method of nutrition, nutritional support recommendations, and peri-operative nutrition) were chosen based on the items identified by practitioners dealing with malnutrition in children with CHD in real-world practice, in order to ensure a holistic approach in addressing the areas of uncertainty and provide comprehensive guidance for dealing with CHD from a nutritional aspect. The final version of the survey questions is available in Supplementary File 1.

### 2.3 Consensus criteria

We utilized an online survey tool (Lime Survey) to gather the panelists’ input on each of the two voting waves. A 5-point Likert scale was used to rate statements by each panelist. Each individual vote was considered as agreement if either “agree” or “strongly agree” was selected. The rest of the points (i.e., neutral, disagree, and strongly disagree) were considered as opposing votes. A threshold of 80% consensus was selected for this consensus process, with consensus defined as either ≥80% agreement or ≥80% disagreement with a statement. The results were reported as percentages of agreement.

### 2.4 Modified Delphi procedure

An initial online survey was used to gather insights and feedback from the first group of experts on the initial version of the statements. One-to-one interviews were held with each of the Group 1 panelists to individually discuss non-consensus votes and allow the panel to raise comments on any of the statements. These interviews were then followed by an online group workshop, in which the statements were revised and discussed to eliminate any disagreements. The comments made by the panelists during this workshop were then considered in the discussion section. The second wave of the Delphi process included an online survey in which the second group of experts participated anonymously. Group 2 panelists who voted neutrally on critical statements received follow-up calls or emails to clarify their positions. A *post-hoc* refinement round was added to address disagreements. The final voting results, and in turn the inclusion of statements, were determined based on the pooled votes of the 15 panel members on the finalized statements, where statements that reached the threshold for consensus (i.e., 80% or more) upon adding the results of both panel groups were included in the final consensus document. Figure 1 illustrates the modified Delphi process.

**Figure 1:**
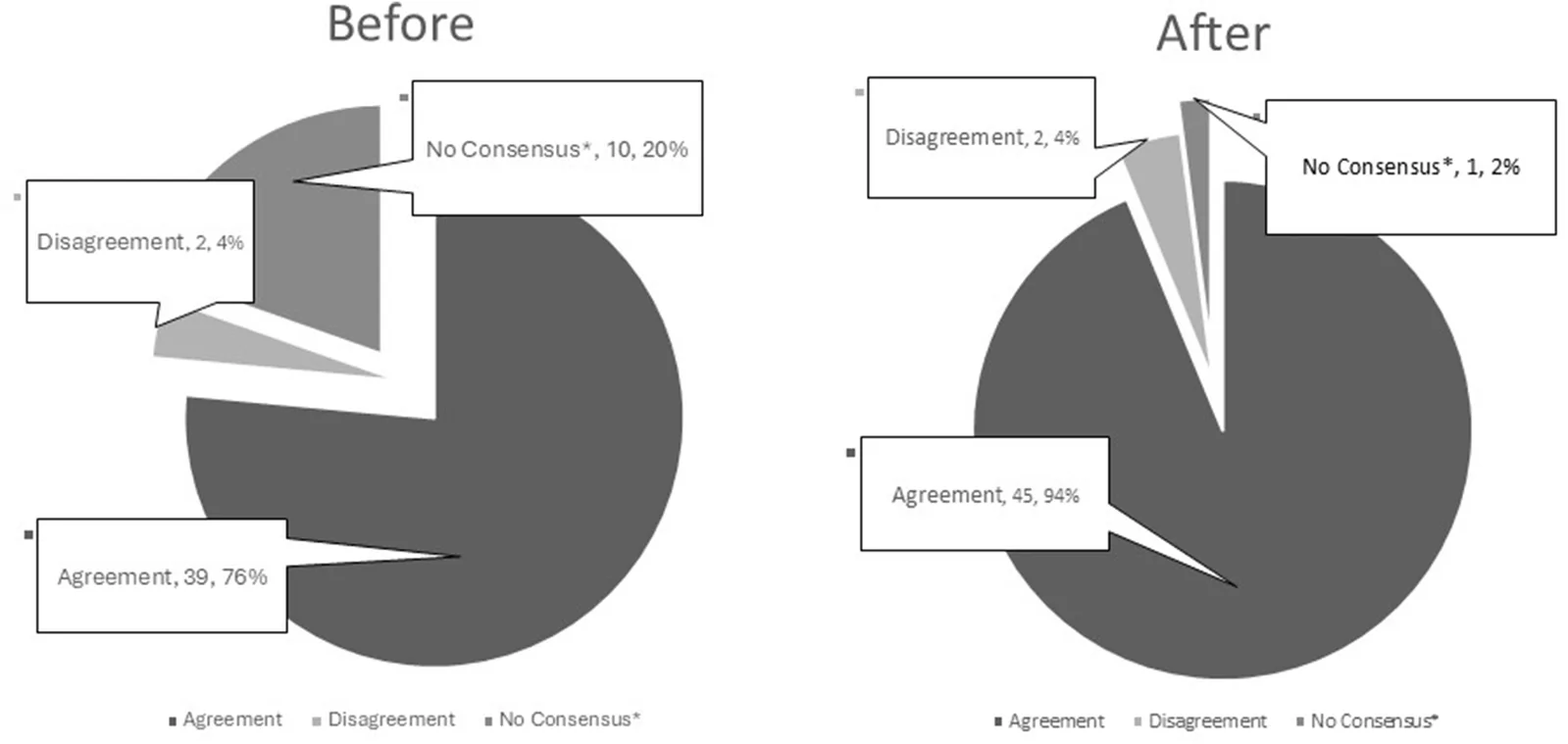
Stepwise modified Delphi approach adopted in the present work.

## 3 Results

### 3.1 Panel demographics and response rates

Fifteen panelists completed the Delphi process. In two of the 48 statements, one panelist (Group 2) abstained from voting due to the lack of qualifications needed to develop an opinion on the matter. Analysis of the final voting results was completed in December 2024. Our panel included practitioners from the UAE (40%, *n* = 6), Egypt (26.7%, *n* = 4), Kuwait (20%, *n* = 3), and KSA (13.3%, *n* = 2). Pediatric cardiologists represented one third of the panel (*n* = 5), nutritionists/dietitians represented 20% (*n* = 3), and the rest of the panel (*n* = 7) were either pediatricians or neonatologists.

### 3.2 Delphi wave 1

Wave 1 online voting took place in July 2024 and was succeeded by an online expert workshop that took place in September 2024. Edits, including adding, removing, or rephrasing, were applied to ten statements based on comments raised by Group 1 panelists during one-to-one meetings held after the initial voting (Table 1). The workshop was concluded by reaching a positive consensus on 45 of the 48 statements (93.8%), with two of the remaining statements (4.10.B and 4.11.B) meeting the consensus threshold of 80% with the disagreement votes. A comparison between agreement degrees before and after the expert workshop is illustrated in Figure 2, with ‘’no consensus’’ representing the statements that did not reach the agreed 80% threshold of agreement or disagreement.

**Table 1:** Changes to Delphi statements after Wave 1 workshop.

Section	Initial statement	Vi%	Edits	V% *N* = 5	Final status V% *N* = 15
1	1.1. The pathologies below could have risk of nutritional impact on CHD:	60%	1.1 The pathologies below could have risk of nutritional impact on CHD:	100%	100%
	Congestive heart failure—Cyanotic heart disease—Pulmonary hypertension—Single ventricle septal defects—Coarctation of the aorta -Pulmonary Stenosis/Aortic stenosis Cardiomyopathy (e.g.: ventricular hypertrophy, etc.)		Congestive heart failure—Cyanotic heart disease—Pulmonary hypertension—Single ventricle septal defects—Coarctation of the aorta -Pulmonary stenosis/Aortic stenosis—Cardiomyopathy		
			1.2. The pathologies below could have high risk of nutritional impact on CHD:	100%	100%
			Hemodynamically significant acyanotic congenital heart diseases (e.g.: septal defects, Coarctation of the aorta, etc.)—Congestive heart failure—Cyanotic congenital heart disease—Pulmonary hypertension		
2	NA	NA	2.7. ECG is one of the cardiac tools that should be used during a full cardiac assessment	100%	92.9%^a^
3	3.6.1. Nutrition through breastfeeding doesn’t differ in impact between cyanotic and acyanotic patients	40%	3.6. Breastfeeding in high-risk congenital heart disease may not provide sufficient energy support without supportive high energy-dense formula	100%	80%
	3.6.2 Breastfeeding pattern is less in CHD infants comparable to healthy infants due to mother-infant periods of separation and clinical manifestations	80%			
4	4.0. A term ill newborn initially needs 40–60 kcal/kg/day, with a gradual increase to 90–120 kcal/kg/day. The daily requirement of carbohydrates is between 9–14 g/kg/day (40%–50% of total calories), proteins— 1.8–2.2 g/kg/day (7%–16% of total calories), and lipids—of 4–6 g/kg/day (34%–35% of total calories).	20%	Removed	NA	NA
	4.1. In neonates with a CHD, the daily caloric intake should be up to 50% higher, but without exceeding 150 mL/kg/day liquid volume, and daily protein requirements can be as high as 3 g/kg/day.	100%	4.1. In neonates with a CHD, the daily caloric intake should be up to 50% higher, but without exceeding 150 mL/kg/day liquid volume	100%	100%
	4.7.1. Sodium intake recommended range is from 0—3 mmol/kg/Day	60%	4.7. Electrolytes intake is determined according to recommended reference; in cases of lab results-proven electrolytes imbalance, treating physicians should provide supportive treatments for correction of serum electrolyte levels.	100%	93.3%
	4.7.2. Calcium intake recommended range is from 0.25–1.5 mmol/kg/Day^1^	60%			
	4.7.3. Potassium intake recommended range is from 0.8–1.5 mmol/kg/Day^1^	60%			
	4.7.4. Chloride intake recommended range is from 0—5 mmol/kg/Day^1^	60%			
	4.9 Regarding the needs of infants with CHD in critical care:	100%	4.9. Regarding the needs of infants with CHD Post-surgery:	100%	86.7%
	Energy: Determined by indirect calorimetry, if available, resting energy expenditure (∼55–60 kcal/kg) in the first 3–5 days after surgery or until CRP is <2 mg/dL		Energy: Determined by indirect calorimetry, if available, resting energy expenditure (∼55–60 kcal/kg) in the first 3–5 days after surgery or until CRP is <2 mg/dL		
	Protein:		Proteins:		
	Term: 3–3.5 g/kg		Term: 3-3.5 g/kg		
	Preterm or LBW: 3-4 g/kg		Preterm or LBW: 3-4 g/kg		
	Fluid:		Fluids:		
	As per critical care fluid restriction (generally 50%–80% MIVF with liberalization after medication wean and sternal closure		As per critical care fluid restriction (generally 50%–80% MIVF with liberalization after medication wean and sternal closure)		
	4.10. For the following patient, the appropriate nutrition strategy should be: Not growing well e.g.1**-**2 centile below birth centile, CHD lesion with higher nutrition risk but drinks well, finished > 75% of feeds orally, Fluid intake < 120 mL/kg/day for actions (1, 2) and methods (A,B,C):				
	1- Approximately 10% extra energy 100–110 kcal/kg (protein contributing 9%–12% energy) 2- Approximately 30–50% extra protein around 2.5 g/kg protein	100%	1- Approximately 10% extra energy 100–110 kcal/kg (protein contributing 9%–12% energy) 2- Approximately 30%–50% extra protein around 2.5 g/kg protein	100%	93.3%
	A. Combination of breastfeeding and high energy-dense formula	80%	A. Combination of breastfeeding and high energy-dense formula	100%	86.7%
	B. Combination of standard formula and high energy-dense formula	20%	B. Combination of standard formula and high energy-dense formula	20%	46.7%
	C. high-energy dense formula	40%	C. In case of breastfeeding unavailability, high energy-dense Formula usage alone	100%	NA^b^
	4.11. For the following patients, the appropriate nutrition strategy should be: Not growing e.g., >2 centile below birth centile, CHD lesion with a higher nutrition risk, Requires NGT/NJT for feeding, Fluid intake < 100 mL/kg/day for actions (1, 2) and methods (A,B,C):				
	1- Approximately 10% extra energy 100–110 kcal/kg (protein contributing 9%–12% energy) 2- Approximately 30%–50% extra protein around 2.5 g/kg protein)	80%	1- Approximately 10% extra energy 100–110 kcal/kg (protein contributing 9%–12% energy) 2- Approximately 30%–50% extra protein around 2.5 g/kg protein)	100%	93.3%
	A. Combination of breastfeeding and high energy-dense formula	80%	A. Combination of breastfeeding and high energy-dense formula	80%	86.7%
	B. Combination of standard formula and high energy-dense formula	0%	B. Combination of standard formula and high energy-dense formula	0%	33.3%
	C. high-energy dense formula	60%	C. In case of breastfeeding unavailability, high energy-dense formula usage alone	100%	100%
	NA	NA	4.12. The high energy-dense formula can safely increase nutrient intake and promote weight gain in CHD infants. This nutritional formulation could therefore potentially prevent malnutrition and failure to thrive in CHD infants	100%	100%

CHD, Congenital Heart Disease; ECG, electrocardiogram; CRP, C-reactive protein; LBW, low birth weight; MIVF, maintenance intravenous fluids; NGT, nasogastric tube; NJT, Nasojejunal Tube; Vi%, initial voting percentage of agreement; V%, final voting percentage of agreement; NA, not applicable.

a One panelist chose to not vote instead of giving a neutral vote so the total number of votes was 14.

b This statement was removed after the *post-hoc* round described in subsection 3.3; the final vote results on the updated version is available in Table 3.

**Figure 2:**
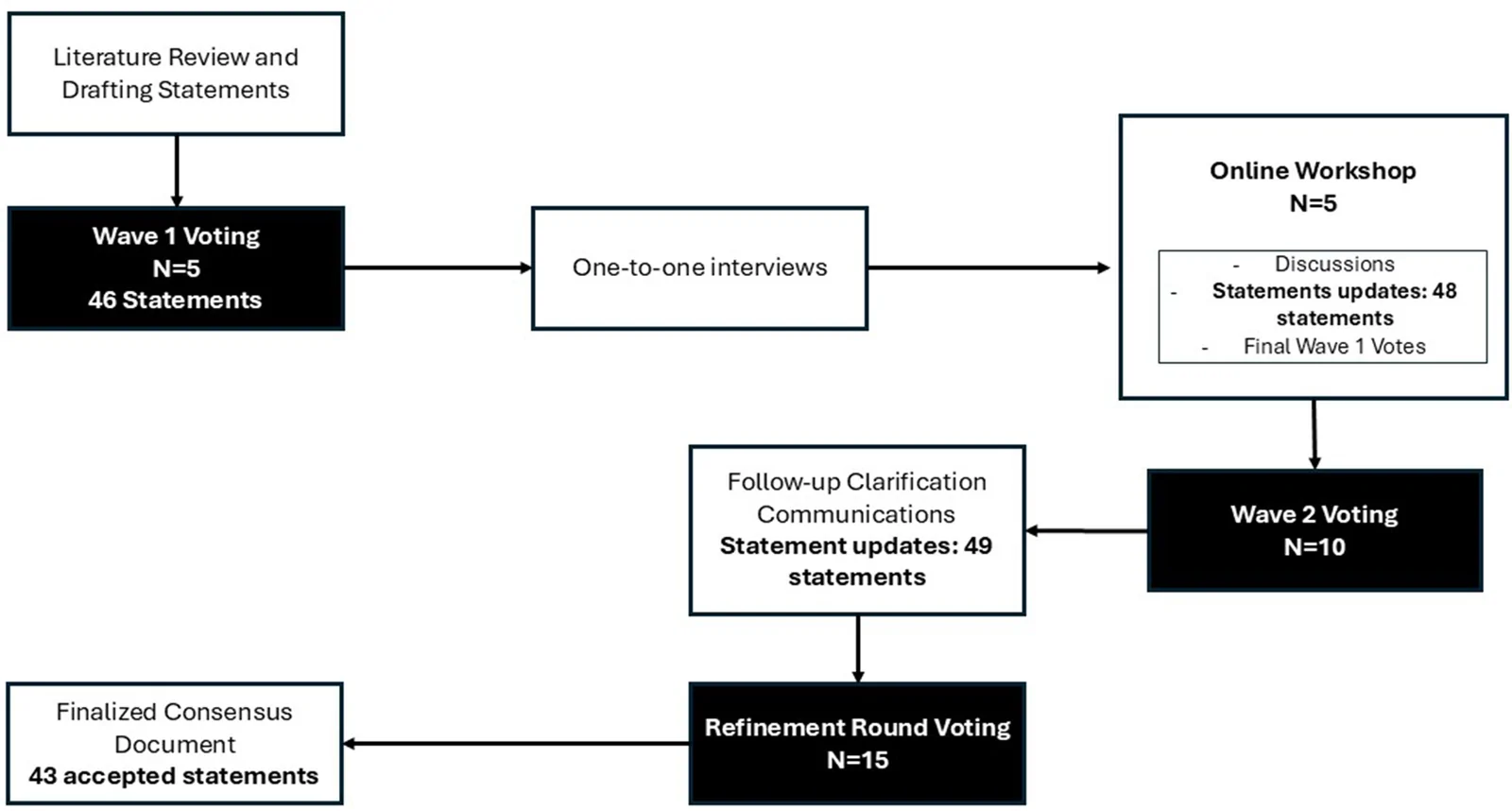
Summary of changes to the statements after Wave 1 workshop. Figure 2 legend: *No Consensus: statements did not reach the agreed 80% threshold of agreement or disagreement.

### 3.3 Delphi wave 2

Wave 2 online survey voting took place from October 2024 to November 2024. It resulted in 40 of 48 statements (83.3%) reaching consensus. After contacting the panelists who voted “neutral” on the remaining critical statements for further clarification and discussion, all the neutral votes on these statements were resolved, and the percentage of consensus increased to 89.6%, i.e., 43 of 48 statements. Table 2 summarizes the changes to votes after follow-up communications. All of the 43 statements had 80% or more agreement, i.e., only positive consensus was achieved.

**Table 2:** Follow-up communications to wave 2 neutral votes and the resulting changes.

Statement no.	Initial			Final			Consensus reached (final response)
	+	−	*n*	+	−	*n*	
2.7	7	1	2	8	1	0^a^	Yes
2.8	7	1	2	7	3	0	No
4.3	6	1	3	7	3	0^a^	No
4.6	7	0	3	10	0	0	Yes
4.9	6	1	3	8	2	0	Yes

+: agreement, −: disagreement, *n*: neutral vote.

a One panelist chose to not vote instead of giving a neutral vote.

### 3.4 *Post-hoc* refinement round

Following the scheduled rounds, qualitative feedback from the panel indicated that the options laid down by the statements did not cover the current recommendations and practices adopted in real-world settings. Consequently, an additional statement was added to address this limitation, and the panelists (Groups 1 and 2) were contacted via email and phone calls to complete another round of voting on the two relevant statements. Table 3 describes the edits introduced in this round as well as the subsequent shifts in the votes.

**Table 3:** Final edits made during the *post-hoc* refinement round and final voting results.

	Round 2 (*N* = 15)		Additional round (final votes; *N* = 15)	
4	Statement	V%	Statement	V%
	4.10. For the following patient, the appropriate nutrition strategy should be: Not growing well e.g.1–2 centile below birth centile, CHD lesion with higher nutrition risk but drinks well, finished >75% of feeds orally, Fluid intake <120 mL/kg/day for actions (1, 2) and methods (A, B, C):			
	1- Approximately 10% extra energy 100–110 kcal/kg (protein contributing 9%–12% energy) 2- Approximately 30%–50% extra protein around 2.5 g/kg protein	93.3%	1- Approximately 10% extra energy 100–110 kcal/kg (protein contributing 9%–12% energy) 2- Approximately 30%–50% extra protein around 2.5 g/kg protein	93.3%
	A. Combination of breastfeeding and high energy-dense formula	86.7%	A. Combination of breastfeeding and high energy-dense formula	86.7%
	B. Combination of standard formula and high energy-dense formula	46.7%	B. Combination of standard formula and high energy-dense formula	46.7%
	C. In case of breastfeeding unavailability, high energy-dense Formula usage alone?	100%	C. In case of breastfeeding unavailability, high energy-dense formula usage alone	53.3%
			D. In case of breastfeeding unavailability, standard formula in addition to 30%–80% high energy-dense formula usage	66.7%

### 3.5 Overall results

After compiling the results for both waves, in addition to the *post-hoc* round, consensus was reached on 43 (87.8%) statements, which comprised the final statements to be included in this consensus document (Supplementary File 2). The Peri-operative Nutrition section received the highest degree of agreement among other sections, with a mean of 96.4% agreement across the section statements. On the other hand, the fourth section, Nutritional Support Recommendations, had the lowest mean degree of agreement (77.9%).

Regarding nutritional risk in CHD, the panel agreed on the classification of CHD types according to the risk of malnutrition, clinical presentations of malnutrition in CHD, and other factors contributing to CHD burden in malnourished infants. Regarding nutritional assessment of infants with CHD, the panel agreed on the criteria and cut-off points for malnutrition in these patients, factors that should be assessed for patient history, as well as laboratory tests and investigations to be performed for a complete nutritional assessment. For methods of nutrition, the panel specified values for fluid intake and macronutrient intake in enteral and parenteral feeding, respectively, as well as the role of breastfeeding in infants with CHD. The panel also agreed on different aspects of nutritional support, including recommended caloric intake, fluid intake, management of electrolytes and fluids, nutrition in acute and critical care, and the use of high-density formula. The detailed statements and the percentage of agreement are listed in Supplementary File 3.

## 4 Discussion

This consensus document introduces the cornerstones for nutritional assessment and management for infants with CHD in four Middle Eastern countries, aiming to address the care pathways of malnourished infants with CHD, starting from their early assessment to post-operative nutritional support and follow-up. The utilization of both evidence from the literature and suggestions from clinical experts allowed for establishing the connection between real-world clinicians’ practices and the published literature.

The risk of malnutrition associated with CHD is well-documented. However, several factors are associated with a higher risk of malnutrition. In our consensus process, we focused on CHD-related conditions that pose an additional risk for inadequate nutrition. Our panel identified congestive heart failure, cyanotic heart disease, and pulmonary hypertension as major risk factors affecting nutritional state, which aligns with findings from prior studies (5, 23, 24). Our panel also agreed that only hemodynamically significant septal defects, aortic and pulmonary stenosis, and coarctation of the aorta are associated with an increased risk of malnutrition; i.e., the degree of anatomical abnormality and its impact on blood flow and cardiac function are what govern their impact on the risk of malnutrition. These factors have been previously debated to determine the nutritional risk they pose (22), where the multifactorial nature of malnutrition in CHD necessitates a comprehensive assessment to determine the actual nutritional risk upon CHD diagnosis.

Regarding referral to nutritionists, our panelists acknowledged that these infants are ideally managed by a multidisciplinary team (MDT) that includes a nutritionist; however, in case of MDT unavailability, it is recommended to refer newborns with CHD to a pediatric nutritionist when a delay in growth is evident despite following nutritional support recommendations. Another consensus document preferred to base referrals on the total risk of malnutrition, endured by cardiac and extracardiac factors (22); however, this consensus recommendation reflects the regional practices and should be interpreted in this context. On the other hand, early referral should be considered for its favorable outcomes in this population (21).

Assessments in infants with CHD should include cardiac history, nutritional history, in addition to general parameters, e.g., socioeconomic information and family history (25); our panelists listed the items to be considered for comprehensive patient reports (Supplementary File 2). Genetic predisposition to inadequate or delayed growth should be assessed, as well as other factors known to affect growth, to adjust growth targets individually (7, 19). The panelists agreed that growth parameters should be regularly assessed in all follow-up visits, with the time intervals between such visits determined based on the patient's status and the degree of malnutrition, to range between weekly visits in complicated cases to monthly visits in more stable cases. It is important to note that increasing the frequency of follow-up visits has shown favorable outcomes regarding growth improvement in patients with CHD (21).

The panelists’ comments, in addition to items obtaining non-consensus results in wave 1 voting, have uncovered possible discrepancies between recommendations adapted from various literature and practice in the Middle East. For instance, our panelists agreed that indirect calorimetry should be used—when available—to determine the exact energy needs for infants in post-surgical critical care; however, all the panelists indicated that indirect calorimetry is rarely used in practice, i.e., its use is restricted to research and academic purposes. While indirect calorimetry is considered a gold standard for assessing energy expenditure in critically ill infants (26, 27), resting energy expenditure (REE) can also be calculated using predictive equations (28, 29). This is particularly relevant in the case of infants with CHD, as they are usually smaller than average with unstable hemodynamics, which may pose a limitation to indirect calorimetry use (28, 30). Additionally, limited financial resources and the lack of trained professionals may also limit the use of indirect calorimetry in this case. Our panelists agreed that using methods other than indirect calorimetry in calculating REE does not negatively impact the course of recovery of those infants.

Similarly, while literature provides recommendations for electrolyte daily intake in infants with CHD (29) insights from practice opt for a less proactive approach, where electrolyte administration is recommended by the panelists only in cases where patients present—after being administered feeding formulae with fixed electrolyte concentrations—with symptoms suggestive of electrolyte imbalance, or whenever follow-up laboratory investigations show electrolyte imbalance. Additionally, electrolyte intake in these patients is determined in a case-by-case manner, where concomitant medications (e.g., diuretics), the patient's status, and laboratory results govern the recommended concentrations.

Nutritional support in infants with CHD showing signs of suboptimal growth despite adequate intake [i.e., breastfeeding or standard formula with normal drinking abilities or nasojejunal [NJT] or nasogastric tube [NGT]] remains challenging (30). The panel agreed that a high-energy-dense formula is recommended in case breastfeeding is not available; however, we failed to reach consensus on whether it should be used alone or in combination with standard formula at a level of up to 80%. This variability in expert opinions suggests that nutritional management approaches for these infants can vary across the region. In another consensus document on nutritional pathways for these cases, a high-energy-dense formula was recommended in combination with a standard formula or on its own, according to the patient's growth assessment (21). Our panelists explained that in practice, some cases require a very high caloric intake, e.g., prior to surgeries and in cases of high protein intake, which warrants the use of a high-energy-dense formula in proportions approaching 100% of nutrition requirements. The panelists also highlighted the importance of tailoring interventions to the patient's needs. For instance, these infants are usually familiar with their original feeding method; accordingly, replacing only three feeds with high-energy-dense formula can be optimal to ensure the gradual introduction of the new formula. In such cases, patients’ growth parameters are then monitored closely to determine whether the remaining feeds are also to be replaced with high-energy-dense formula. Given that these unresolved areas reflect a genuine uncertainty and variability in current clinical practice, further research is warranted to inform clinical recommendations regarding similar cases, in addition to further characterizing the rationale behind such variability.

Gradual increase of fluid intake is recommended with enteral feeding until the desired intake of 150–180 mL/kg/day is reached (27). However, the rate at which enteral feeding is increased primarily depends on the patient's tolerance. Complications such as respiratory issues, infections, and gastroesophageal reflux (GER), in addition to chromosomal abnormalities and birth complications, usually slow down the enteral feeding process. Caloric intake is similarly regulated in terms of daily increase in both enteral and parenteral nutrition, where the highest tolerable caloric intake is to be administered, with concomitant monitoring for potential side effects arising from calories or volume intake. Consequently, progressive weight gain and meeting energy targets in shorter periods of time require close monitoring and adjustments when applicable (27). The panelists agreed that infants with heart failure, cardiomyopathy, pulmonary hypertension, and other high nutritional risk conditions are indicated for high caloric intake (i.e., 175–180 kcal/kg/day) with lower fluid volumes, to compensate for the increased energy expenditure associated with these conditions.

Our panelists supported the use of high-energy-dense formula in infants with CHD, where manually fortifying standard formula was often sought prior to the introduction of high-energy-dense formula. Accordingly, it is recommended to educate clinicians on its indications and guidelines for use. We also identified other existing gaps in malnutrition management in infants with CHD in the Middle East. Delayed CHD diagnosis was one of the most common issues, where patients usually present to a pediatric nutritionist prior to having an established cardiac diagnosis or treatment. This pattern was evident in a retrospective study in Egypt in 2021, where 13% of the enrolled infants with CHD had “failure to thrive” as their initial presentation upon CHD diagnosis (31). There is also a lack of awareness among HCPs on essential management approaches; for instance, adjustments for renal function preservation with high-protein intake and indications for high-energy-dense formula are largely overlooked.

Our consensus document has the inherent limitations of the Delphi consensus process (32), including heavily relying on the opinions of clinicians in formulating statements, in addition to the bias arising from face-to-face, non-anonymous discussion workshops, which may shift opinions towards a single direction. In our consensus process, we ensured that all the inputs of our steering committee (Group 1) were gathered individually in one-to-one meetings to minimize the impact of this type of bias. Nevertheless, post-voting communications for clarification of neutral votes might have introduced interviewer or conformity bias to our process; moreover, a *post-hoc* refinement round was introduced after the planned Delphi rounds, which may have introduced further bias by allowing new response options to emerge. However, we considered this modification necessary to accurately capture real-world practices that were not adequately represented by the original response options. Additionally, despite meeting the threshold of the Delphi approach for the sample size (33), the small sample size (*N* = 15) participating in our work may have limited the variability of opinions, and in turn, fewer areas of non-consensus were uncovered. Another limitation would be that this consensus document did not comprehensively address different targets and thresholds for all clinical scenarios, which necessitates the use of the established recommendations alongside individualized patient assessment. Our work can also have a relatively limited generalizability in Middle Eastern countries with no representation in our panel; however, this consensus document can act as a foundation for localized protocols addressing minor differences in these regions.

In conclusion, this consensus document serves as a foundational guide for improving the nutritional care of infants with CHD in the Middle East. By bridging the gap between evidence-based recommendations and real-world clinical practice, we provided a detailed description of recommended assessment and management practices in this population. Future efforts should focus on addressing the identified gaps, localizing these guidelines to establish country-specific protocols, and enhancing adherence to these recommendations among healthcare providers to ensure that all infants with CHD receive the comprehensive care they need.
