# Supplementary material for: Neonatal and pediatric nutritional care: a modified Delphi consensus on dietary management for infants with congenital heart disease in four Middle Eastern countries
Source: Front Pediatr. 2026 Sep 30;14:1881464. doi: 10.3389/fped.2026.1881464 (PMC13661917; doi:10.3389/fped.2026.1881464)
Supplement: Supplementary file 1 [file Supplementaryfile1.pdf]

Nutritional Management of Congenital Heart Disease Infants

Congenital heart disease (CHD) in infants is a complex condition that can significantly impact the lives of infants affecting their proper nutrition which plays a vital role in their growth, development, and overall health. However, the specific dietary needs of children with CHD may vary depending on the severity of their condition and other contributing factors.

Despite the existence of international guidelines, there is a significant gap in consensus in the Middle East region due to the unique dietary needs, cultural practices, and socioeconomic contexts. Thus Danone, has led a Regional Delphi-consensus to establish standardized practices to ensure consistent and optimal nutritional care, ultimately improving patient outcomes among healthcare professionals in the Middle East.

Danone is privileged to your participation in such an important consensus where all your valuable insights and clinical expertise are highly regarded for the effective management of congenital heart disease in middle eastern infants.
There are 46 questions in this survey.

Introduction

Could you please provide your name? *

Please write your answer here:

Could you please specify your specialty? *

Please write your answer here:

1. CHD Nutritional Risk

1.1 The below pathologies could have risk of nutritional impact on CHD:

- Congestive heart failure
- Cyanotic Heart Disease
- pulmonary hypertension
- Single ventricle
- Septal defects
- coarctation of the aorta
- Pulmonary Stenosis/ Aortic Stenosis
- Cardiomyopathy (e.g.: ventricular hypertrophy, etc…)

*

Please choose **only one** of the following:

- ◯ Strongly Disagree
- ◯ Disagree
- ◯ Neutral
- ◯ Agree
- ◯ Strongly Agree

1.2 The below pathologies could have high risk of nutritional impact on CHD:

- Hemodynamically significant acyanotic congenital heart diseases (e.g.: septal defects, coarctation of the aorta, etc…)
- Congestive Heart Failure
- Cyanotic Congenital heart disease
- Pulmonary Hypertension

*

Please choose **only one** of the following:

◯ Strongly Disagree
◯ Disagree
◯ Neutral
◯ Agree
◯ Strongly Agree

1.3 The below forms of malnutrition are present in CHD patients:

- Wasting
- Stunting
- Underweight
- Thinness

*

Please choose **only one** of the following:

◯ Strongly Disagree
◯ Disagree
◯ Neutral
◯ Agree
◯ Strongly Agree

1.4 Genetic mutations* play a critical role in CHD occurrence

*such as DNA methylation mutations, BMPR1A mutations, mutations in transcription factors, chromosomal abnormalities, etc.…

*

Please choose **only one** of the following:

◯ Strongly Disagree
◯ Disagree
◯ Neutral
◯ Agree
◯ Strongly Agree

1.5 The below clinical associations increase CHD burden:

- Intrauterine growth restriction
- Respiratory infections
- GERD
- Diarrhea
- Anorexia
- Chronic Hypoxia

*

Please choose **only one** of the following:

◯ Strongly Disagree
◯ Disagree
◯ Neutral
◯ Agree
◯ Strongly Agree

2. CHD Nutritional Assessment

2.1 The criteria for determining malnutrition in a pediatric CHD patient are:

- Weight for age < 3rd percentile
- Height/Length for age < 3rd percentile
- Weight for Length < 3rd percentile
- Reduction in >2 percentiles for weight for age, length/height for age, and/or weight for length

*

Please choose **only one** of the following:

◯ Strongly Disagree
◯ Disagree
◯ Neutral
◯ Agree
◯ Strongly Agree

2.2 The below factors should be assessed separately or merged for a complete patient history assessment:

- Type and severity of cardiac lesion
- Medical and surgical history, including prematurity
- Family history
- Socioeconomic factors, including financial status and family and living situation
- Genetic potential for growth

*

Please choose **only one** of the following:

◯ Strongly Disagree
◯ Disagree
◯ Neutral
◯ Agree
◯ Strongly Agree

2.3 The below factors should be assessed separately or merged for a complete nutrition-focused physical findings assessment:

- Overall Appearance
- Cardiopulmonary system—increased work of breathing, wet lung sounds
- Extremities, muscles, and bones—loss of subcutaneous fat, muscle wasting, peripheral edema, peripheral cyanosis, muscle tone
- Digestive system—compromised swallow function, difficulty coordinating sucking, swallowing, breathing, abdominal distention or pain, poor appetite, ascites, nausea/vomiting/reflux, diarrhea
- Eyes and head—sunken eyes, sunken fontanelle, hair loss, temporal wasting
- Neurological changes
- Skin—dermatitis, dry/scaly, poor wound healing
- Vital signs—blood pressure, heart rate

*

Please choose **only one** of the following:

◯ Strongly Disagree
◯ Disagree
◯ Neutral
◯ Agree
◯ Strongly Agree

2.4 The below factors should be assessed separately or merged for a complete nutrition related history assessment:

- Breast milk/infant formula intake
- Medications with nutrition-related side effects
- Feeding behaviors, including aversion, refusal, and fatigue
- Adherence to nutrition education/instructions, as proper formula mixing
- Energy, fluid, macronutrient, and micronutrient intake (from all sources, including oral intake, enteral or parenteral nutrition)
- Breastfeeding success/problems
- Food/nutrition program participation
- Vitamin/mineral supplement use
- Enteral/parenteral nutrition intake

*

Please choose **only one** of the following:

◯ Strongly Disagree
◯ Disagree
◯ Neutral
◯ Agree
◯ Strongly Agree

2.5 The below factors should be assessed separately or merged for a complete nutritional assessment:

- Weight for height Z-score
- Age for weight Z-score
- Height for age Z-score
- BMI for age Z-score
- Caloric intake
- Triceps skin fold thickness
- Subscapular skin fold thickness

*

Please choose **only one** of the following:

◯ Strongly Disagree
◯ Disagree
◯ Neutral
◯ Agree
◯ Strongly Agree

2.6 The below cardiac findings should be assessed separately or merged for a complete cardiac assessment:

- Modified Ross score
- X-ray chest and heart
- Echocardiogram

*

Please choose **only one** of the following:

- ◯ Strongly Disagree
- ◯ Disagree
- ◯ Neutral
- ◯ Agree
- ◯ Strongly Agree

2.7 Electrocardiogram (ECG) is one of the cardiac tools that should be used during a full cardiac assessment *

Please choose **only one** of the following:

- ◯ Strongly Disagree
- ◯ Disagree
- ◯ Neutral
- ◯ Agree
- ◯ Strongly Agree

2.8 The below investigations should be assessed separately or merged for proper diagnosis of CHD patients' nutritional status:

- Complete Blood Counts
- HIV DNA PCR
- Pro-BNP
- Arterial oxygen saturation
- Serum Iron and Ferritin
- Serum Albumin
- Liver functions
- Thyroid hormones

*

Please choose **only one** of the following:

- ◯ Strongly Disagree
- ◯ Disagree
- ◯ Neutral
- ◯ Agree
- ◯ Strongly Agree

3. Recommendations Based on Method of Nutrition

3.1 Regarding enteral nutrition for newborns with CHD, these values are recommended starting day 1:

a- Day 1: Minimal enteral feeding: 10-20ml/kg/day

b- Increase daily to reach (only when possible): 150 – 180 mL/kg/d (close monitoring of NEC signs above 100 mL/kg/day)
*

Please choose **only one** of the following:

- Strongly Disagree
- Disagree
- Neutral
- Agree
- Strongly Agree

3.2 Enteral nutrition in terms of caloric intake for infants with CHD should be the following:

a- Term: 100 – 120 kcal/kg/day

b- Preterm: 110 – 135 kcal kg/day (Up to 175 – 180 kcal/kg/day depending on the type of CHD)
*

Please choose **only one** of the following:

- Strongly Disagree
- Disagree
- Neutral
- Agree
- Strongly Agree

3.3 Regarding parenteral nutrition for newborns with CHD, these values are recommended starting day 1:

- **Glucose:**
  a- Term: 6 g/kg/day
  b- Preterm: 8 g/kg/day
- **Proteins**
  a- Term: 1 g/kg/day
  b- Preterm: 1.5 g/kg/day
- **Lipids**
  a- Term: 1 g/kg/day
  b- Preterm: 1 g/kg/day

*

Please choose **only one** of the following:

- Strongly Disagree
- Disagree
- Neutral
- Agree
- Strongly Agree

3.4 Regarding parenteral nutrition for newborns with CHD, these values are recommended starting day 1:

- **Energy Intake:**
  a- Term: 85-95 kcal/kg/day
  b- Preterm: 85-105 kcal/kg/day
- **Glucose:**
  a- Term: 6 g/kg/day
  b- Preterm: 8 g/kg/day
- **Proteins:**
  a- Term: 1 g/kg/day
  b- Preterm: 1.5 g/kg/day
- **Lipids:**
  a- Term: 1 g/kg/day
  b- Preterm: 1 g/kg/day

*

Please choose **only one** of the following:

◯ Strongly Disagree
◯ Disagree
◯ Neutral
◯ Agree
◯ Strongly Agree

3.5 Breastfeeding is encouraged due to its protection in term, preterm, and infants undergoing surgery *

Please choose **only one** of the following:

◯ Strongly Disagree
◯ Disagree
◯ Neutral
◯ Agree
◯ Strongly Agree

3.6 Breastfeeding in high-risk congenital heart disease may not provide sufficient energy support without supportive high energy-dense formula *

Please choose **only one** of the following:

◯ Strongly Disagree
◯ Disagree
◯ Neutral
◯ Agree
◯ Strongly Agree

4. Nutritional Support Recommendations

4.1 In neonates with a CHD, the daily caloric intake should be up to 50% higher, but without exceeding 150 mL/kg/day liquid volume *

Please choose **only one** of the following:

- Strongly Disagree
- Disagree
- Neutral
- Agree
- Strongly Agree

4.2 Calorie intake need may vary from 130–150 kcal/kg/day up to 180 kcal/kg/day according to the type of CHD *

Please choose **only one** of the following:

- Strongly Disagree
- Disagree
- Neutral
- Agree
- Strongly Agree

4.3 Energy Intake in acute phase of CHD:

Energy intake should not exceed the resting energy expenditure in acute phase to avoid a negative impact on cardiac output and After acute phase, energy intake is based on resting energy expenditure, physical activity, rehabilitation, and growth
*

Please choose **only one** of the following:

- Strongly Disagree
- Disagree
- Neutral
- Agree
- Strongly Agree

4.4 Energy-enriched and protein-enriched formulas provide higher nutrients intake than standard-formula *

Please choose **only one** of the following:

- Strongly Disagree
- Disagree
- Neutral
- Agree
- Strongly Agree

4.5 Fluid Intake recommendations:

Using diuretics and strict fluid restriction are recommended, Fluid intake in term infants is 60–70 mL/kg on day 1 and increases to 100–120 mL/kg by day 2 or 3, Premature infants may receive 70–80 mL/kg on day 1 and slowly advance to 150 mL/kg/day
*

Please choose **only one** of the following:

- ◯ Strongly Disagree
- ◯ Disagree
- ◯ Neutral
- ◯ Agree
- ◯ Strongly Agree

4.6 The minimum amount of protein in CHD patients to avoid a negative nitrogen balance is 1.5 g/kg/day, and the maximum intake of amino acids should not exceed 3 g/kg/day *

Please choose **only one** of the following:

- ◯ Strongly Disagree
- ◯ Disagree
- ◯ Neutral
- ◯ Agree
- ◯ Strongly Agree

4.7 Electrolytes intake is determined according to recommended reference and in cases of lab results-proven electrolytes imbalance, HCPs provide supportive treatments for correction of serum electrolyte levels *

Please choose **only one** of the following:

- ◯ Strongly Disagree
- ◯ Disagree
- ◯ Neutral
- ◯ Agree
- ◯ Strongly Agree

4.8 Regarding the needs of infants with CHD in step-down/acute care:

- **Energy:**
  - 120–150 kcal/kg; **140–200** kcal/kg for catch-up growth*
  *Catch-up growth equation (kcal/kg): Kcal/Kg for weight X ideal weight)/ current weight
- **Proteins:**
  a- Term: 3-3.5 g/kg
  b- Preterm or LBW: 3-4 g/kg
- **Fluids:**
  a- <3 kg: 120 mL/kg
  b- >3 kg: 100 mL/kg

  - Consider +10–15% to compensate for increased losses with tachypnea, diarrhea, emesis, and diuresis

*

Please choose **only one** of the following:

◯ Strongly Disagree
◯ Disagree
◯ Neutral
◯ Agree
◯ Strongly Agree

4.9 Regarding the needs of infants with CHD Post-surgery:

- **Energy:**
  Determined by indirect calorimetry, if available resting energy expenditure (~55–60 kcal/kg) in the first 3–5 days after surgery or until CRP is <2 mg/dl
- **Proteins:**
  a- Term: 3-3.5 g/kg
  b- Preterm or LBW: 3-4 g/kg
- **Fluids**:
  As per critical care fluid restriction (generally 50–80% MIVF with liberalization after medication wean and sternal closure)

*

Please choose **only one** of the following:

◯ Strongly Disagree
◯ Disagree
◯ Neutral
◯ Agree
◯ Strongly Agree

4.10 For the following patient, the appropriate nutrition strategy should be:

Not growing well e.g.1-2 centile below birth centile, CHD lesion with higher nutrition risk but drinks well, finished >75% of feeds orally, Fluid intake < 120ml/kg/day

Action:

1- Approximately 10% extra energy 100 – 110 kcal/kg (protein contributing 9-12% energy)

2- Approximately 30 – 50% extra protein around 2.5g/kg protein)

*

Please choose the appropriate response for each item:

	Strongly Disagree	Disagree	Neutral	Agree	Strongly Agree
**Combination of breastfeeding and high energy-dense formula**	◯	◯	◯	◯	◯
**Combination of standard formula and high energy-dense formula**	◯	◯	◯	◯	◯
**In case of breastfeeding unavailability, High Energy-Dense Formula usage alone**	◯	◯	◯	◯	◯

4.10.a Do you agree with the actions taken in the above Patient case? *

Please choose **only one** of the following:

- ◯ Strongly Disagree
- ◯ Disagree
- ◯ Neutral
- ◯ Agree
- ◯ Strongly Agree

4.11 For the following patient, the appropriate nutrition strategy should be:

Not growing e.g. >2 centile below birth centile, CHD lesion with a higher nutrition risk, Requires NGT/NJT for feeding, Fluid intake < 100ml/kg/day

Action:

1- Approximately 10% extra energy 100 – 110 kcal/kg (protein contributing 9-12% energy)

2- Approximately 30 – 50% extra protein around 2.5g/kg protein)

*

Please choose the appropriate response for each item:

	Strongly Disagree	Disagree	Neutral	Agree	Strongly Agree
**Combination of breastfeeding and high energy-dense formula**	◯	◯	◯	◯	◯
**Combination of standard formula and high energy-dense formula**	◯	◯	◯	◯	◯
**In case of breastfeeding unavailability, High Energy-Dense Formula usage alone**	◯	◯	◯	◯	◯

4.11.a Do you agree with the actions taken in the above patient case? *

Please choose **only one** of the following:

◯ Strongly Disagree

◯ Disagree

◯ Neutral

◯ Agree

◯ Strongly Agree

4.12 The high energy-dense formula can safely increase nutrient intake and promote weight gain in CHD infants. This nutritional formulation could therefore potentially prevent malnutrition and failure to thrive in CHD infants *

Please choose **only one** of the following:

◯ Strongly Disagree

◯ Disagree

◯ Neutral

◯ Agree

◯ Strongly Agree

5. Recommendations on Peri-Operative Nutrition

5.1 Regarding infants with CHD in the Pre-operative neonatal period:

Adequate nutrition should be provided (preferably via EN) to meet the patient's needs until surgery *

Please choose **only one** of the following:

- Strongly Disagree
- Disagree
- Neutral
- Agree
- Strongly Agree

5.2 Regarding the needs of infants with CHD in post-operative critical care:

- Nutrition support should be initiated as soon as possible to prevent development/worsening of malnutrition, minimize the loss of lean body mass, and to support functioning of vital organs
- The refeeding syndrome should be avoided in infants with significant malnutrition
- Overfeeding should be avoided, which can cause difficulty in weaning from the ventilator
- Unnecessary cessation of enteral nutrition should be reduced

*

Please choose **only one** of the following:

- Strongly Disagree
- Disagree
- Neutral
- Agree
- Strongly Agree

5.3 Regarding the needs of infants with CHD in post-discharge/ Home-Setting period:

Should demonstrate age-appropriate or catch-up weight gain and growth and should demonstrate age-appropriate feeding behaviors
*

Please choose **only one** of the following:

- Strongly Disagree
- Disagree
- Neutral
- Agree
- Strongly Agree

5.4 The Use of Energy Enriched Formula is associated with decreased rate of side effects and antibiotic use after surgery *

Please choose **only one** of the following:

- ◯ Strongly Disagree
- ◯ Disagree
- ◯ Neutral
- ◯ Agree
- ◯ Strongly Agree

5.5 High Energy Nutrition could shorten ICU stay, decrease ventilator support time and reduce post-operative weight loss

*

Please choose **only one** of the following:

- ◯ Strongly Disagree
- ◯ Disagree
- ◯ Neutral
- ◯ Agree
- ◯ Strongly Agree

5.6 Parenteral nutrition is recommended in CHD infants in early post-operative stage or mechanically ventilated or suffer from gastrointestinal issues *

Please choose **only one** of the following:

- ◯ Strongly Disagree
- ◯ Disagree
- ◯ Neutral
- ◯ Agree
- ◯ Strongly Agree

5.7 Administration of early high energy-dense feeding to infants after congenital heart surgery is associated with improved growth, reduced CICU stay, decreased ventilator support time, and reduced postoperative infection rates

*

Please choose **only one** of the following:

- ◯ Strongly Disagree
- ◯ Disagree
- ◯ Neutral
- ◯ Agree
- ◯ Strongly Agree

5.8 Regarding the needs of infants with CHD in step-down/acute care:

- Adequate nutrition should be provided to meet needs and correct nutrient or electrolyte deficiency.
- Transition from parenteral nutrition to 100% enteral nutrition.
- Transition enteral nutrition to oral or combination oral/supplemental enteral nutrition; develop enteral nutrition or oral schedule appropriate for home.
- Demonstrate age-appropriate or catch-up weight gain and growth
- Parents/caregivers should demonstrate competency in delivering appropriate nutrition prior to discharge

*

Please choose **only one** of the following:

◯ Strongly Disagree
◯ Disagree
◯ Neutral
◯ Agree
◯ Strongly Agree

5.9 Early high energy-dense feeding to infants post-discharge is associated with a higher malnutrition recovery rate *

Please choose **only one** of the following:

◯ Strongly Disagree
◯ Disagree
◯ Neutral
◯ Agree
◯ Strongly Agree

5.10 The use of energy enriched formula is safe and beneficial and may be used for feeding in children younger than 2 years undergoing CHD surgery *

Please choose **only one** of the following:

◯ Strongly Disagree
◯ Disagree
◯ Neutral
◯ Agree
◯ Strongly Agree

5.11 High energy-dense formula, when compared to standard formula, demonstrates superior nutrient intake and nitrogen balance in infants following congenital heart surgery, leading to improved growth parameters such as weight, height, and mid-arm circumference and their z-scores post discharge *

Please choose **only one** of the following:

- ◯ Strongly Disagree
- ◯ Disagree
- ◯ Neutral
- ◯ Agree
- ◯ Strongly Agree

Thank you for your precious time and insights.

Submit your survey.
Thank you for completing this survey.
