# Supplementary material for: Neonatal and pediatric nutritional care: a modified Delphi consensus on dietary management for infants with congenital heart disease in four Middle Eastern countries
Source: Front Pediatr. 2026 Sep 30;14:1881464. doi: 10.3389/fped.2026.1881464 (PMC13661917; doi:10.3389/fped.2026.1881464)
Supplement: Supplementary file 2 [file Supplementaryfile2.docx]

1. **CHD Nutritional Risk**
   1.1. Pathologies that could have a risk of nutritional impact on congenital heart disease (CHD) are congestive heart failure, cyanotic heart disease, pulmonary hypertension, a single ventricle, septal defects, coarctation of the aorta, pulmonary stenosis/aortic stenosis, and cardiomyopathy (e.g., ventricular hypertrophy, etc.)
   1.2. Pathologies that could have a high risk of nutritional impact on CHD are hemodynamically significant acyanotic congenital heart diseases (e.g., septal defects, coarctation of the aorta, etc.), congestive heart failure, cyanotic congenital heart disease, and pulmonary hypertension.
   1.3. Wasting, stunting, underweight, and thinness, are the forms of malnutrition present in CHD patients.
   1.4. Genetic mutations — such as DNA methylation mutations, BMPR1A mutations, mutations in transcription factors, and chromosomal abnormalities, etc. —play a critical role in CHD occurrence.
   1.5. The clinical associations that increase CHD burden are intrauterine growth restriction, respiratory infections, gastroesophageal reflux disease , diarrhea, anorexia, and chronic hypoxia.
2. **Nutritional Assessment**
   2.1. The criteria for determining malnutrition in a pediatric CHD patient are:

- Weight for age <3rd percentile
- Height/Length for age <3rd percentile
- Weight for Length <3rd percentile
- A decline of >2 percentiles for weight for age, length/height for age, and/or weight for length
  2.2. The factors that should be assessed — separately or merged — for a complete patient history assessment are:
- Type and severity of the cardiac lesion
- Medical and surgical history, including prematurity
- Family history
- Socioeconomic factors, including financial status and family and living situation
- Genetic potential for growth
  2.3. The factors that should be assessed — separately or merged — for a complete nutrition-focused physical findings assessment:
- Overall appearance
- Cardiopulmonary system: increased breathing work and wet lung sounds
- Extremities, muscles, and bones: loss of subcutaneous fat, muscle wasting, peripheral edema, peripheral cyanosis, and muscle tone
- Digestive system: compromised swallow function, difficulty coordinating sucking, swallowing, breathing, abdominal distention or pain, poor appetite, ascites, nausea/vomiting/reflux, and diarrhea
- Eyes and head: sunken eyes, sunken fontanelle, hair loss, and temporal wasting
- Neurological changes
- Skin: dermatitis, dry/scaly, and poor wound healing
- Vital signs: blood pressure and heart rate
  2.4. The factors that should be assessed — separately or merged — for a complete nutrition-related history assessment are:
- Breast milk/infant formula intake
- Medications with nutrition-related side effects
- Feeding behaviors, including aversion, refusal, and fatigue
- Adherence to nutrition education/instructions, e.g., proper formula mixing,
- Energy, fluid, macronutrient, and micronutrient intake (from all sources, including oral intake, enteral or parenteral nutrition)
- Breastfeeding success/problems
- Food/nutrition program participation
- Vitamin/mineral supplement use
- Enteral/parenteral nutrition intake
  2.5. The factors that should be assessed — separately or merged — for a complete nutritional assessment are:
- Weight for height Z-score
- Age for weight Z-score
- Height for age Z-score
- Body mass index (BMI) for age Z-score
- Caloric intake
- Triceps skin fold thickness
- Subscapular skin fold thickness
  2.6. Cardiac findings that should be assessed — separately or merged — for a complete cardiac assessment are:
- Modified Ross score
- Chest X-ray
- Echocardiography
  2.7. Electrocardiogram is one of the cardiac tools that should be used during a full cardiac assessment.
  2.8. Investigations that should be assessed — separately or merged — for a proper diagnosis of CHD patients' nutritional status are:
- Complete blood count (CBC)
- HIV DNA PCR
- N-terminal pro-B-type natriuretic peptide (NT-proBNP)
- Arterial oxygen saturation
- Serum iron and ferritin
- Serum albumin
- Liver function tests
- Thyroid function tests

3. **Method of Nutrition**
   3.1. Regarding enteral nutrition for newborns with CHD, recommended values are:

- Day 1: minimal enteral feeding (10-20ml/kg/day)
- Enteral feeding is increased daily (only when possible) to reach 150-180 mL/kg/day (with close monitoring of necrotizing enterocolitis signs when more than 100 mL/kg/day is reached)
  3.2. Enteral nutrition in terms of caloric intake for infants with CHD should be:
- Term: 100-120 kcal/kg/day
- Preterm: 110-135 kcal/kg/day (Up to 175-180 kcal/kg/day depending on the type of CHD)
  3.3. Regarding parenteral nutrition for newborns with CHD, the recommended values for macronutrients are:

Glucose:

- Term: 6 g/kg/day
- Preterm: 8 g/kg/day

Proteins:

- Term: 1 g/kg/day
- Preterm: 1.5 g/kg/day

Lipids:

- Term: 1 g/kg/day
- Preterm: 1 g/kg/day
  3.4. Regarding parenteral nutrition for newborns with CHD, the recommended values are:

Energy intake:

- Term: 85-95 kcal/kg/day
- Preterm: 85-105 kcal/kg/day

Glucose:

- Term: 6 g/kg/day
- Preterm: 8 g/kg/day

Proteins:

- Term: 1 g/kg/day
- Preterm: 1.5 g/kg/day

Lipids:

- Term: 1 g/kg/day
- Preterm: 1 g/kg/day
  3.5. Breastfeeding is encouraged due to its protection in term, preterm, and infants undergoing surgery.
  3.6. Breastfeeding in high-risk congenital heart disease may not provide sufficient energy support without supportive high energy-dense formula.

4. **Nutritional Support Recommendations**
   4.1. In neonates with a CHD, the daily caloric intake should be increased by up to 50%, without exceeding 150 mL/kg/day liquid volume.
   4.2. Calorie intake needs may vary from 130 to 150 kcal/kg/day, up to 180 kcal/kg/day according to the type of CHD.
   4.3. Energy-enriched and protein-enriched formulas provide higher nutrients intake than standard-formula.
   4.4. Fluid Intake recommendations:

- Diuretics and strict fluid restriction are recommended
- Fluid intake in term infants is 60-70 mL/kg on Day 1, to be increased to 100-120 mL/kg by Day 2 or Day 3
- Premature infants may receive 70-80 mL/kg on Day 1 and slowly advance to 150 mL/kg/day.
  4.5. The minimum amount of protein in CHD patients to avoid a negative nitrogen balance is 1.5 g/kg/day, and the maximum intake of amino acids should not exceed 3 g/kg/day.
  4.6. Electrolytes intake is determined according to recommended reference; in cases of lab results-proven electrolytes imbalance, treating physicians should provide supportive treatments for correction of serum electrolyte levels.
  4.7. Regarding the needs of infants with CHD in step-down/acute care:

Energy:

- 120-150 kcal/kg and 140-200 kcal/kg for catch-up growth

Proteins:

- Term: 3-3.5 g/kg
- Preterm or low birth weight (LBW): 3-4 g/kg

Fluids:

- <3 kg: 120 mL/kg
- >3 kg: 100 mL/kg (considering +10-15% to compensate for increased losses in cases of tachypnea, diarrhea, emesis, and diuresis)
  4.8. Regarding the needs of infants with CHD Post-surgery:

Energy (determined by indirect calorimetry, if available):

- Resting energy expenditure (about 55-60 kcal/kg) in the first 3-5 days after surgery or until the C-reactive protein concentration is <2 mg/dl

Proteins:

- Term: 3-3.5 g/kg
- Preterm or LBW: 3-4 g/kg

Fluids:

- As per critical care fluid restriction (generally 50%-80% maintenance intravenous fluids with liberalization after medication weaning and sternal closure)
  4.9. The high energy-dense formula can safely increase nutrient intake and promote weight gain in CHD infants. This nutritional formulation could therefore potentially prevent malnutrition and failure to thrive in CHD infants.
  4.10. For patients that are not growing well, e.g.,1-2 centile below birth centile, have a CHD lesion with higher nutrition risk but drink well, finished > 75% of feeds orally, with a fluid intake <120ml/kg/day, the appropriate nutrition strategy should be:
- Approximately 10% extra energy (100-110 kcal/kg, with protein contributing to 9-12% of energy)
- Approximately 30%-50% extra protein (i.e., around 2.5g/kg protein)
- Combination of breastfeeding and high energy-dense formula
  4.11. For patients with failure to growth, e.g., >2 centile below birth centile, with a CHD lesion with a higher nutrition risk, who require nasojejunal/nasogastric tube (NGT/NJT) for feeding, and a fluid intake < 100ml/kg/day, the appropriate nutrition strategy should be:
- Approximately 10% extra energy (100-110 kcal/kg, with protein contributing to 9-12% of energy)
- Approximately 30%-50% extra protein (i.e., around 2.5g/kg protein)
- Combination of breastfeeding and high energy-dense formula, and in case of breastfeeding unavailability, high energy-dense formula usage alone.

5. **Peri Operative Nutrition**
   5.1. Regarding infants with CHD in the preoperative neonatal period, adequate nutrition should be provided (preferably via enteral nutrition) to meet the patient's needs until surgery.
   5.2. Regarding the needs of infants with CHD in postoperative critical care:

- Nutrition support should be initiated as soon as possible to prevent development/worsening of malnutrition, minimize the loss of lean body mass, and to support functioning of vital organs.
- Refeeding syndrome should be avoided in infants with significant malnutrition.
- Overfeeding should be avoided, which can cause difficulty in weaning from the ventilator.
- Unnecessary cessation of enteral nutrition should be reduced.
  5.3. Regarding the needs of infants with CHD in the post-discharge/home-setting period:
- Infants should demonstrate age-appropriate or catch-up weight gain and growth.
- Infants should demonstrate age-appropriate feeding behaviors.
  5.4. The use of energy enriched formula is associated with a decreased rate of side effects and antibiotic use after surgery.
  5.5. high energy nutrition could shorten ICU stay, decrease ventilator support time, and reduce post-operative weight loss.
  5.6. Administration of early high energy-dense feeding to infants after congenital heart surgery is associated with improved growth, reduced cardiac ICU stay, decreased ventilator support time, and reduced postoperative infection rates.
  5.7. Regarding the needs of infants with CHD in step-down/acute care:
- Adequate nutrition should be provided to meet the infant’s needs and correct any nutrient or electrolyte deficiency.
- Infants should transition from parenteral nutrition to 100% enteral nutrition.
- Infants should transition from enteral nutrition to oral or a combination of oral and supplemental enteral nutrition, and an enteral nutrition or oral schedule appropriate for home should be developed.
- Infants should demonstrate age-appropriate or catch-up weight gain and growth.
- Parents/caregivers should demonstrate competency in delivering appropriate nutrition prior to discharge.
  5.8. Early high energy-dense feeding to infants post-discharge is associated with a higher malnutrition recovery rate.
  5.9. The use of energy enriched formula is safe and beneficial and may be used for feeding in children younger than two years undergoing CHD surgery.
  5.10. High energy-dense formula, when compared to standard formula, demonstrates superior nutrient intake and nitrogen balance in infants following congenital heart surgery, leading to improved growth parameters such as weight, height, and mid-arm circumference and their Z-scores post discharge.
