# Supplementary material for: Neonatal and pediatric nutritional care: a modified Delphi consensus on dietary management for infants with congenital heart disease in four Middle Eastern countries
Source: Front Pediatr. 2026 Sep 30;14:1881464. doi: 10.3389/fped.2026.1881464 (PMC13661917; doi:10.3389/fped.2026.1881464)
Supplement: Supplementary file 3 [file Supplementaryfile3.docx]

**Supplementary File 3: Final Delphi results**

| **section** | **Final Statements** | **References** | **Final vote**  **N=15** |
| --- | --- | --- | --- |
| **1** | 1.1 **Pathologies below could have a risk of nutritional impact on CHD:**   Congestive heart failure - cyanotic heart disease - pulmonary hypertension - a single ventricle - septal defects - coarctation of the aorta - pulmonary stenosis/aortic stenosis - cardiomyopathy | (1–6) | 100% |
|  | 1.2. **Pathologies below could have a high risk of nutritional impact on CHD:**   Hemodynamically-significant acyanotic congenital heart diseases (e.g., septal defects, coarctation of the aorta, etc.) - congestive heart failure - cyanotic congenital heart disease - pulmonary hypertension |  | 100% |
|  | 1.3. **The forms of malnutrition below are present in CHD patients:**   Wasting - stunting - underweight - thinness | (1,7) | 100% |
|  | 1.4. Genetic mutations (such as DNA methylation mutations, BMPR1A mutations, mutations in transcription factors, chromosomal abnormalities, etc.) play a critical role in CHD occurrence | (8,9) | 86.7% |
|  | 1.5. **The below clinical associations increase CHD burden:**   Intrauterine growth restriction - respiratory infections - GERD - diarrhea - anorexia - chronic hypoxia | (5,10–13) | 93.3% |
| **2** | **2.1. The criteria for determining malnutrition in a pediatric CHD patient are:**  weight for age <3^rd^ percentile - height/length for age <3^rd^ percentile - weight for length <3^rd^ percentile – decline of >2 percentiles for weight for age, length/height for age, and/or weight for length | (14) | 93.3% |
|  | **2.2. The following factors should be assessed separately or merged for a complete patient history assessment:**  Type and severity of cardiac lesion - medical and surgical history including prematurity - family history - socioeconomic factors including financial status and family and living situation - genetic potential for growth | (14) | 93.3% |
|  | **2.3. The following factors should be assessed separately or merged for a complete nutrition-focused physical findings assessment:**  Overall appearance - cardiopulmonary system (increased work of breathing, wet lung sounds) - extremities (loss of subcutaneous fat, muscle wasting, peripheral edema, peripheral cyanosis, muscle tone) - digestive system (compromised swallow function, difficulty coordinating sucking, swallowing, breathing, abdominal distention or pain, poor appetite, ascites, nausea/vomiting/reflux, diarrhea) - eyes and head (sunken eyes, sunken fontanelle, hair loss, temporal wasting) - neurological changes - skin (dermatitis, dry/scaly, poor wound healing) - vital signs (blood pressure, heart rate) | (14) | 86.7% |
|  | **2.4. The following factors should be assessed separately or merged for a complete nutrition-related history assessment:**  Breast milk/infant formula intake - medications with nutrition-related side effects - feeding behaviors, including aversion, refusal, and fatigue - adherence to nutrition education/instructions, such as proper formula mixing - energy, fluid, macronutrient, and micronutrient intake (from all sources, including oral intake, enteral, or parenteral nutrition) - breastfeeding success/problems - food/nutrition program participation - vitamin/mineral supplement use - enteral/parenteral nutrition intake | (14) | 93.3% |
|  | **2.5. The following factors should be assessed separately or merged for a complete nutritional assessment:**  Weight for height Z-score - age for weight Z-score - height for age Z-score - BMI for age Z-score - caloric intake - triceps skin fold thickness - subscapular skin fold thickness | (5,7,15) | 93.3% |
|  | **2.6. The below cardiac tools should be assessed separately or merged for a complete cardiac assessment:**  Modified Ross score – chest X-ray - echocardiography | (7,16) | 93.3% |
|  | 2.7. ECG is one of the cardiac tools that should be used during a full cardiac assessment | NA | 92.9%* |
|  | **2.8. The investigations below should be assessed separately or merged for proper diagnosis of CHD patients’ nutritional status:**  CBC - HIV DNA PCR testing – ProBNP - arterial oxygen saturation - serum iron and ferritin - serum albumin - liver function tests - thyroid function tests | (7,16–19) | 80% |
| **3** | **3.1. Regarding enteral nutrition for newborns with CHD, these values are recommended starting day 1**:  Day 1: minimal enteral feeding (10-20ml/kg/day)  Enteral feeding is increased daily (only when possible) to reach 150-180 mL/kg/day (with close monitoring of necrotizing enterocolitis signs when more than 100 mL/kg/day is reached) | (6) | 86.7% |
|  | **3.2.** **Enteral nutrition in terms of caloric intake for infants with CHD should be the following**:  Term: 100 – 120 kcal/kg/day  Preterm: 110 – 135 kcal kg/day (Up to 175 – 180 kcal/kg/day depending on the type of CHD) | (6) | 93.3% |
|  | **3.3.** **Regarding parenteral nutrition for newborns with CHD, these values are recommended starting from Day 1**:  Glucose:  Term: 6 g/kg/day - Preterm: 8 g/kg/day  Proteins:  Term: 1 g/kg/day - Preterm: 1.5 g/kg/day  Lipids:  Term: 1 g/kg/day - Preterm: 1 g/kg/day | (6) | 93.3% |
|  | **3.4.** **Regarding parenteral nutrition for newborns with CHD, these values are recommended starting from Day 1**:  Energy Intake:  Term: 85-95 kcal/kg/day - Preterm: 85-105 kcal/kg/day  Glucose:  Term: 6 g/kg/day - Preterm: 8 g/kg/day  Proteins:  Term: 1 g/kg/day - Preterm: 1.5 g/kg/day  Lipids:  Term: 1 g/kg/day - Preterm: 1 g/kg/day | (6) | 93.3% |
|  | 3.5. Breastfeeding is encouraged due to its protection in term, preterm, and infants undergoing surgery | (6) | 100% |
|  | 3.6. Breastfeeding in high-risk congenital heart disease may not provide sufficient energy support without supportive high energy-dense formula | (12) | 80% |
|  |  |  |  |
| **4** | 4.1. In neonates with a CHD, the daily caloric intake should be up to 50% higher, but without exceeding 150 mL/kg/day liquid volume | (20) | 100% |
|  | 4.2. Calorie intake need may vary from 130 to 150 kcal/kg/day and up to 180 kcal/kg/day according to the type of CHD | (6) | 100% |
|  | **4.3.** **Energy Intake in acute phase of CHD:**  Energy intake should not exceed the resting energy expenditure in acute phase to avoid a negative impact on cardiac output and after the acute phase, energy intake is based on resting energy expenditure, physical activity, rehabilitation, and growth | (20) | 78.6%* |
|  | 4.4. Energy-enriched and protein-enriched formulas provide higher nutrients intake than standard-formula | (21) | 100% |
|  | **4.5.** **Fluid Intake recommendations**:  Using diuretics and strict fluid restriction are recommended  Fluid intake in term infants is 60–70 mL/kg on Day 1, to be increased to 100–120 mL/kg by Day 2 or 3  Premature infants may receive 70–80 mL/kg on day 1 and slowly advance to 150 mL/kg/day | (20) | 93.3% |
|  | 4.6. The minimum amount of protein in CHD patients to avoid a negative nitrogen balance is 1.5 g/kg/day | (20) | 100% |
|  | 4.7. Electrolytes intake is determined according to recommended reference; in cases of lab results-proven electrolytes imbalance, treating physicians should provide supportive treatments for correction of serum electrolyte levels. | NA | 93.3% |
|  |  |  |  |
|  |  |  |  |
|  |  |  |  |
|  | **4.8.** **Regarding the needs of infants with CHD in step-down/acute care**:  Energy:  120–150 kcal/kg - 140–200 kcal/kg for catch-up growth**  Proteins:  Term: 3-3.5 g/kg - Preterm or LBW: 3-4 g/kg  Fluids:  <3 kg: 120 mL/kg - >3 kg: 100 mL/kg (considering +10–15% to compensate for increased losses in cases tachypnea, diarrhea, emesis, and diuresis) | (14) | 86.7% |
|  | **4.9. Regarding the needs of infants with CHD post-surgery:**  Energy (determined by indirect calorimetry, if available):  resting energy expenditure (about 55–60 kcal/kg) in the first 3–5 days after surgery or until CRP is <2 mg/dl  Proteins:  Term: 3-3.5 g/kg - Preterm or LBW: 3-4 g/kg  Fluids:  As per critical care fluid restriction (generally 50–80% MIVF with liberalization after medication weaning and sternal closure) | (14) | 86.7% |
|  | **4.10. For the following patient: not growing well, e.g., 1-2 centile below birth centile, CHD lesion with higher nutrition risk but drinks well, finished >75% of feeds orally, Fluid intake <120ml/kg/day, the appropriate nutrition strategy should be:** | | |
|  | 1. Approximately 10% extra energy (100–110 kcal/kg; protein contributing to 9-12% of energy) 2. Approximately 30%–50% extra protein around 2.5g/kg protein | (22) | 93.3% |
|  | A. Combination of breastfeeding and high energy-dense formula |  | 86.7% |
|  | B. Combination of standard formula and high energy-dense formula |  | 46.7% |
|  | C. In case of breastfeeding unavailability, high energy-dense formula usage alone |  | 53.3% |
|  | D. In case of breastfeeding unavailability, standard formula in addition to 30-80% high energy-dense  formula usage |  | 66.7% |
|  | **4.11. For the following patient: not growing, e.g., >2 centile below birth centile, CHD lesion with a higher nutrition risk, requires NGT/NJT for feeding, fluid intake <100ml/kg/day, , the appropriate nutrition strategy should be:** | | |
|  | 1. Approximately 10% extra energy (100–110 kcal/kg; protein contributing to 9-12% of energy) 2. Approximately 30%–50% extra protein around 2.5g/kg protein | (22) | 93.3% |
|  | A. Combination of breastfeeding and high energy-dense formula |  | 86.7% |
|  | B. Combination of standard formula and high energy-dense formula |  | 33.3% |
|  | C. In case of breastfeeding unavailability, high energy-dense formula usage alone |  | 100% |
|  | 4.12. The high energy-dense formula can safely increase nutrient intake and promote weight gain in CHD infants. This nutritional formulation could therefore potentially prevent malnutrition and failure to thrive in CHD infants | (23) | 100% |
| **5** | 5.1 Regarding infants with CHD in the preoperative neonatal period: adequate nutrition should be provided (preferably via enteral feeding) to meet the patient’s needs until surgery | (14) | 100% |
|  | **5.2**. **Regarding the needs of infants with CHD in postoperative critical care**  Nutrition support should be initiated as soon as possible to prevent development/worsening of malnutrition, minimize the loss of lean body mass, and to support functioning of vital organs.  Refeeding syndrome should be avoided in infants with significant malnutrition.  Overfeeding should be avoided, which can cause difficulty in weaning from the ventilator.  Unnecessary cessation of enteral nutrition should be reduced. | (14) | 100% |
|  | **5.3 Regarding the needs of infants with CHD in post-discharge/home-setting period**  Infants should demonstrate age-appropriate or catch-up weight gain and growth and should demonstrate age-appropriate feeding behaviors. | (14) | 100% |
|  | 5.4 The use of energy enriched formula is associated with decreased rate of side effects and antibiotic use after surgery | (14) | 93.3% |
|  | 5.5 High energy nutrition could shorten ICU stay, decrease ventilator support time and reduce post-operative weight loss | (24) | 100% |
|  | 5.6 Parenteral nutrition is recommended for CHD infants in early postoperative stage, for those requiring mechanical ventilation, or for those with gastrointestinal dysfunction. | (25) | 66.7% |
|  | 5.7 Administration of early high energy-dense feeding to infants after congenital heart surgery is associated with improved growth, reduced cardiac ICU stay, decreased ventilator support time, and reduced postoperative infection rates | (6) | 100% |
|  | **5.8.** **Regarding the needs of infants with CHD in step-down/acute care**:  Adequate nutrition should be provided to meet the infant’s needs and correct any nutrient or electrolyte deficiency.  Infants should transition from parenteral nutrition to 100% enteral nutrition.  Infants should transition from enteral nutrition to oral or a combination of oral and supplemental enteral nutrition, and an enteral nutrition or oral schedule appropriate for home should be developed.  Infants should demonstrate age-appropriate or catch-up weight gain and growth.  Parents/caregivers should demonstrate competency in delivering appropriate nutrition prior to discharge. | (25) | 100% |
|  | 5.9. Early high energy-dense feeding to infants post-discharge is associated with a higher malnutrition recovery rate | (24,25) | 100% |
|  | 5.10. The use of energy enriched formula is safe and beneficial and may be used for feeding in children younger than two years undergoing CHD surgery | (24,25) | 100% |
|  | 5.11. High energy-dense formula, when compared to standard formula, demonstrates superior nutrient intake and nitrogen balance in infants following congenital heart surgery, leading to improved growth parameters such as weight, height, and mid-arm circumference and their Z-scores post discharge | (26) | 100% |

CHD: Congenital Heart Disease, GERD: Gastroesophageal Reflux Disease, CBC: Complete Blood Count, ECG: Electrocardiogram, ICU: Intensive Care Unit, NGT: Nasogastric Tube, NJT: Nasojejunal Tube, LBW: Low Birth Weight, CRP: C-Reactive Protein, MIVF: Maintenance Intravenous Fluids, DNA: Deoxyribonucleic Acid, BMPR1A: Bone Morphogenetic Protein Receptor Type 1A, HIV: Human Immunodeficiency Virus, PCR: Polymerase Chain Reaction, Pro-BNP: Pro-Brain Natriuretic Peptide, BMI: Body Mass Index.

NA: Reference is not available as this statement was developed during the discussion workshop based on the panel’s clinical expertise.

*The total number of votes was 14.

**Catch-up growth equation (kcal/kg): Kcal/Kg for weight X ideal weight)/ current weight.

**References:**

1.	Zhang M, Wang L, Huang R, Sun C, Bao N, Xu Z. Risk factors of malnutrition in Chinese children with congenital heart defect. BMC Pediatr. BioMed Central Ltd.; 2020;20(1). DOI: 10.1186/s12887-020-02124-7

2.	Ismail SR, Mehmood A, Rabiah N, Abu-sulaiman RM, Kabbani MS. Impact of the nutritional status of children with congenital heart diseases on the early post-operative outcome. Gaz Egypt Paediatr Assoc. Springer Science and Business Media LLC; 2021;69(1). DOI: 10.1186/s43054-021-00077-9

3.	Weston C, Husain SA, Curzon CL, Neish S, Kennedy GT, Bonagurio K, et al. Improving Outcomes for Infants with Single Ventricle Physiology through Standardized Feeding during the Interstage. Nurs Res Pract. Hindawi Limited; 2016;2016:1–7. DOI: 10.1155/2016/9505629

4.	Arodiwe I, Chinawa J, Ujunwa F, Adiele D, Ukoha M, Obidike E. Nutritional status of congenital heart disease (CHD) patients: Burden and determinant of malnutrition at university of Nigeria teaching hospital Ituku - Ozalla, Enugu. Pak J Med Sci. 2015;31(5):1140—1145. DOI: 10.12669/pjms.315.6837

5.	Blasquez A, Clouzeau H, Fayon M, Mouton JB, Thambo JB, Enaud R, et al. Evaluation of nutritional status and support in children with congenital heart disease. Eur J Clin Nutr. Nature Publishing Group; 2016;70(4):528–31. DOI: 10.1038/ejcn.2015.209

6.	Salvatori G, De Rose DU, Massolo AC, Patel N, Capolupo I, Giliberti P, et al. Current Strategies to Optimize Nutrition and Growth in Newborns and Infants with Congenital Heart Disease: A Narrative Review. J Clin Med. MDPI; 2022;11(7). DOI: 10.3390/jcm11071841

7.	Batte A, Lwabi P, Lubega S, Kiguli S, Otwombe K, Chimoyi L, et al. Wasting, underweight and stunting among children with congenital heart disease presenting at Mulago hospital, Uganda. BMC Pediatr. BioMed Central Ltd.; 2017;17(1). DOI: 10.1186/s12887-017-0779-y

8.	Joshi RO, Chellappan S, Kukshal P. Exploring the Role of Maternal Nutritional Epigenetics in Congenital Heart Disease. Vol. 4, Current Developments in Nutrition Oxford University Press; 2020.

9.	Trevisan P, Zen TD, Rosa RFM, da Silva JN, Koshiyama DB, Paskulin GA, et al. Chromosomal abnormalities in patients with congenital heart disease. Arq Bras Cardiol. 2013;101(6):495–501. DOI: 10.5935/abc.20130204

10.	Wallenstein MB, Harper LM, Odibo AO, Roehl KA, Longman RE, Macones GA, et al. Fetal congenital heart disease and intrauterine growth restriction: a retrospective cohort study. J Matern Fetal Neonatal Med. Taylor & Francis; 2012;25(6):662–5. DOI: 10.3109/14767058.2011.597900

11.	Jat NK, Bhagwani DK, Bhutani N, Sharma U, Sharma R, Gupta R. Assessment of the prevalence of congenital heart disease in children with pneumonia in tertiary care hospital: A cross-sectional study. Ann Med Surg (Lond). Elsevier Ltd; 2022;73. DOI: 10.1016/j.amsu.2021.103111

12.	Semih Demirtaş M. Approach to Gastroesophageal Reflux: A Cause of Chest Pain in Infants with Congenital Heart Disease. In: Differential Diagnosis of Chest Pain. IntechOpen; 2020. DOI: 10.5772/intechopen.89327

13.	Aufie A, Putra ST, Karyanti MR, Devaera Y. Risk Factors Affecting the Length of Improvement of Nutritional Status in Children with Congenital Heart Disease and Malnutrition. Arch Pediatr Gastr Hepatol Nutr. 2023;2(1):16–22. DOI: https://doi.org/10.58427/apghn.2.1.2023.16-26

14.	Parrish CR, Roman B. Nourishing Little Hearts: Nutritional Implications for Congenital Heart Defects. Pract Gastroenterol [Internet]. 2011;35:11–34. Available from: https://api.semanticscholar.org/CorpusID:74400922

15.	Abdelmoneim HM, Hawary BE, Magdi A, Soliman E. Assessment of Nutrition State in Children with Heart Diseases. Egypt J Hosp Med. 2019;77(2):5049–55.

16.	Shaarawy S, morsy S, Khalifa N, Ahmed A. Comparative Study Between Modified Ross Score and Pediatric Heart Failure Index using pro-BNP in Quantifying Severity of Heart Failure in Pediatrics. Zagazig Univ Med J. Egypts Presidential Specialized Council for Education and Scientific Research; 2020;0(0):0–0. DOI: 10.21608/zumj.2020.30323.1855

17.	Hassan BA, Albanna EA, Morsy SM, Siam AG, Al Shafie MM, Elsaadany HF, et al. Nutritional Status in Children with Un-Operated Congenital Heart Disease: An Egyptian Center Experience. Front Pediatr. Frontiers Media S.A.; 2015;3. DOI: 10.3389/fped.2015.00053

18.	Reiter FP, Hadjamu NJ, Nagdyman N, Zachoval R, Mayerle J, de Toni EN, et al. Congenital heart disease-associated liver disease: A narrative review. Cardiovasc Diagn Ther. AME Publishing Company; 2021;11(2):577–90. DOI: 10.21037/cdt-20-595

19.	Lerner RK, Gruber N, Pollak U. Congenital Heart Disease and Thyroid Dysfunction: Combination, Association, and Implication. World J Pediatr Congenit Heart Surg. SAGE Publications Inc.; 2019;10(5):604–15. DOI: 10.1177/2150135119857704

20.	Luca A-C, Miron IC, Mîndru DE, Curpăn A Ștefania, Stan RC, Țarcă E, et al. Optimal Nutrition Parameters for Neonates and Infants with Congenital Heart Disease. Nutrients. 2022;14(8). DOI: 10.3390/nu14081671

21.	Cui Y, Li L, Hu C, Shi H, Li J, Gupta RK, et al. Effects and Tolerance of Protein and Energy-Enriched Formula in Infants Following Congenital Heart Surgery: A Randomized Controlled Trial. JPEN J Parenter Enteral Nutr. John Wiley and Sons Inc.; 2018;42(1):196–204. DOI: 10.1002/jpen.1031

22.	Marino L V., Johnson MJ, Hall NJ, Davies NJ, Kidd CS, Daniels ML, et al. The development of a consensus-based nutritional pathway for infants with CHD before surgery using a modified Delphi process. Cardiol Young. Cambridge University Press; 2018;28(7):938–48. DOI: 10.1017/S1047951118000549

23.	Taniguchi-Fukatsu A, Matsuoka M, Amagai T. Effect of a high density formula on growth and safety in congenital heart disease infants. E Spen Eur E J Clin Nutr Metab. 2010;5(6):e281–3. DOI: https://doi.org/10.1016/j.eclnm.2010.10.002

24.	Scheeffer VA, Ricachinevsky CP, Freitas AT, Salamon F, Rodrigues FFN, Brondani TG, et al. Tolerability and Effects of the Use of Energy-Enriched Infant Formula After Congenital Heart Surgery: A Randomized Controlled Trial. JPEN J Parenter Enteral Nutr. John Wiley and Sons Inc.; 2020;44(2):348–54. DOI: 10.1002/jpen.1530

25.	Chen X, Zhang M, Song Y, Luo Y, Wang L, Xu Z, et al. Early high-energy feeding in infants following cardiac surgery: A randomized controlled trial. Transl Pediatr. AME Publishing Company; 2021;10(10):2439–48. DOI: 10.21037/tp-21-360

26.	Aryafar M, Mahdavi M, Shahzadi H, Nasrollahzadeh J. Effect of feeding with standard or higher-density formulas on anthropometric measures in children with congenital heart defects after corrective surgery: a randomized clinical trial. Eur J Clin Nutr. 2022;76(12):1713–8. DOI: 10.1038/s41430-022-01186-3
